# RAD52-mediated repair of DNA double-stranded breaks at inactive centromeres leads to subsequent apoptotic cell death

**DOI:** 10.1093/nar/gkae852

**Published:** 2024-10-03

**Authors:** Gen Maruta, Hisanori Maeoka, Toshiyuki Tsunoda, Kozaburo Akiyoshi, Satoshi Takagi, Senji Shirasawa, Shuhei Ishikura

**Affiliations:** Department of Cell Biology, Faculty of Medicine, Fukuoka University, 7-45-1 Nanakuma, Jonan-ku, Fukuoka 814-0180, Japan; Department of Anesthesiology, Faculty of Medicine, Fukuoka University, 7-45-1 Nanakuma, Jonan-ku, Fukuoka 814-0180, Japan; Department of Cell Biology, Faculty of Medicine, Fukuoka University, 7-45-1 Nanakuma, Jonan-ku, Fukuoka 814-0180, Japan; Department of Plastic, Reconstructive and Aesthetic Surgery, Faculty of Medicine, Fukuoka University, 7-45-1 Nanakuma, Jonan-ku, Fukuoka 814-0180, Japan; Department of Cell Biology, Faculty of Medicine, Fukuoka University, 7-45-1 Nanakuma, Jonan-ku, Fukuoka 814-0180, Japan; Center for Advanced Molecular Medicine, Fukuoka University, 7-45-1 Nanakuma, Jonan-ku, Fukuoka 814-0180, Japan; Department of Anesthesiology, Faculty of Medicine, Fukuoka University, 7-45-1 Nanakuma, Jonan-ku, Fukuoka 814-0180, Japan; Department of Plastic, Reconstructive and Aesthetic Surgery, Faculty of Medicine, Fukuoka University, 7-45-1 Nanakuma, Jonan-ku, Fukuoka 814-0180, Japan; Department of Cell Biology, Faculty of Medicine, Fukuoka University, 7-45-1 Nanakuma, Jonan-ku, Fukuoka 814-0180, Japan; Center for Advanced Molecular Medicine, Fukuoka University, 7-45-1 Nanakuma, Jonan-ku, Fukuoka 814-0180, Japan; Department of Cell Biology, Faculty of Medicine, Fukuoka University, 7-45-1 Nanakuma, Jonan-ku, Fukuoka 814-0180, Japan; Center for Advanced Molecular Medicine, Fukuoka University, 7-45-1 Nanakuma, Jonan-ku, Fukuoka 814-0180, Japan

## Abstract

Centromeres, where the kinetochore complex binds, are susceptible to damages including DNA double-stranded breaks (DSBs). Here, we report the functional significance and the temporally and spatially distinct regulation of centromeric DSB repair via the three pathways of non-homologous end joining (NHEJ), homologous recombination (HR) and single-strand annealing (SSA). The SSA factor RAD52 is most frequently recruited to centromeric DSB sites compared with the HR factor RAD51 and the NHEJ factor DNA ligase IV (LIG4), indicating that SSA plays predominant roles in centromeric DSB repair. Upon centromeric DSB induction, LIG4 is recruited to both active centromeres, where kinetochore complex binds, and inactive centromeres. In contrast, RAD51 and RAD52 are recruited only to inactive centromeres. These results indicate that DSBs at active centromeres are repaired through NHEJ, whereas the three pathways of NHEJ, HR and SSA are involved in DSB repair at inactive centromeres. Furthermore, siRNA-mediated depletion of either LIG4 or RAD51 promotes cell death after centromeric DSB induction, whereas RAD52 depletion inhibits it, suggesting that HR and NHEJ are required for appropriate centromeric DSB repair, whereas SSA-mediated centromeric DSB repair leads to subsequent cell death. Thus, SSA-mediated DSB repair at inactive centromeres may cause centromere dysfunction through error-prone repair.

## Graphical Abstract

**Graphical Abstract:**
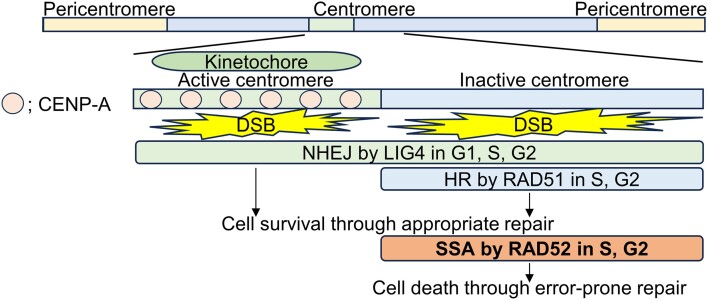

## Introduction

The kinetochore attaches chromosomes to spindle microtubules in mitosis to segregate each sister chromatid into daughter cells. The centromere is an essential chromosomal structure, in which kinetochore protein complex is assembled, to ensure accurate chromosome segregation (1,2). Human centromere is composed of repeats of α-satellite DNA that is 171-bp long and shares 50–80% homology. In each chromosome centromere, a defined number of α-satellite DNA monomers are tandemly organized in a head-to-tail fashion, comprising a larger higher-order repeat (HOR) unit, which is further repeated for several megabases (3). Centromeric regions are functionally divided into active centromeres to which kinetochore complex binds, and the remaining regions called inactive centromeres (4–6). The histone H3 variant CENP-A protein is only deposited into the active centromeres within the centromeres (4–6). Due to the highly repetitive nature of the DNA sequence, the centromere is a fragile genome region where DNA damages are enriched (7). If DNA damage at the centromeres is improperly repaired, dysregulation of the centromere function leads to genome instability and aneuploidy, both of which are frequently observed in many human cancers (8).

DNA double-stranded breaks (DSBs) are one of the most deleterious forms of DNA damage because they cause genome instability and chromosomal translocation (9). DSBs are mainly repaired by two pathways: non-homologous end joining (NHEJ) and homologous recombination (HR) (10). NHEJ is active throughout the cell cycle and involves KU70/KU80-dependent ligation of broken DNA ends by DNA ligase IV (LIG4) (11,12). In contrast, HR occurs in the S and G2 phases of the cell cycle and starts with the binding of the MRN (MRE11/RAD50/NBS1) complex to broken DNA ends to recruit CtIP for short-range DNA end resection (13,14). Further processing of the DNA ends by EXO1, DNA2 and BLM results in the generation of a longer single-stranded DNA (ssDNA) (15). The resulting ssDNA is rapidly bound by replication protein A (RPA) complex (16), which is then replaced by RAD51 to form a nucleoprotein filament that is essential for the subsequent steps of HR (17,18). The choice of pathway for DSB repair is strictly regulated by the cell cycle (19–21), and transcription (22,23) and epigenetic status (24,25) at DSB sites. 53BP1 plays crucial roles in defining DSB repair pathway choice between NHEJ and HR and promotes NHEJ by suppressing HR in cooperation with RIF1 (19,20).

In addition to NHEJ and HR, single-strand annealing (SSA) is adapted to repair DSBs at genomic regions bearing repetitive DNA sequences (26). SSA uses homologous repeats flanking a DSB to join DNA ends. Although there are some mechanistic similarities, SSA and HR are composed of proteins particular to each repair pathway (26). SSA, like HR, requires the long ssDNA, to which RPA binds, but instead of RAD51, the DNA-binding protein RAD52 replaces RPA to coat ssDNA and mediates annealing of DNA that bear homology (27). RAD52-mediated SSA is generally considered to be an error-prone repair pathway as it removes DNA fragments between repeats along with one repeat.

It has been previously reported that centromeric DSBs induced by ionizing radiation are mainly repaired through NHEJ in human cells (28). On the other hand, Yilmaz *et al.* have recently reported that CRISPR-Cas9-mediated DSBs at centromeres are mainly repaired by HR throughout the cell cycle in human and mouse cells (29). Interestingly, they showed that HR factors, including RAD51, BRCA1 and RPA, were recruited to centromeric DSB sites even in the G1 phase, although the recruitments of NHEJ or SSA repair factors were not investigated (29). Furthermore, due to the highly repetitive nature of the DNA sequences, the centromeres are thought to favor SSA for DSB repair. However, roles of SSA in centromeric DSB repair have not been elucidated. Accordingly, the regulatory mechanisms of repair pathway choice for centromeric DSBs remain elusive, even though the centromeres are damage-prone genomic regions, and its dysregulation leads to chromosome instability (30,31).

Here, we established an experimental model in cultured human cells to elucidate the molecular mechanisms of centromeric DSB repair using doxycycline (Dox)-inducible Cas9 (Dox–Cas9) nuclease and centromere-specific sgRNA, and show that the three pathways of HR, SSA and NHEJ are active in centromeric DSB repair. Among these three pathways, SSA plays predominant roles in centromeric DSB repair. We also show that DSBs at active centromeres are mainly repaired through NHEJ throughout the cell cycles, whereas the three pathways of NHEJ, HR and SSA are involved in DSB repair at inactive centromeres in the distinct cell cycle phases. Furthermore, HR and NHEJ are required for appropriate DSB repair at centromeres, whereas SSA-mediated centromeric DSB repair leads to subsequent apoptotic cell death. Thus, these results suggest that SSA-mediated DSB repair at inactive centromeres may cause dysregulation of centromere function through error-prone DSB repair.

## Materials and methods

### Cell culture

HT1080 (ATCC), U2OS (a kind gift from Dr Masatoshi Fujita, Kyushu University, Japan) and Lenti-X 293T (Takara Bio) cells were cultured at 37°C with 5% CO_2_ in Dulbecco’s modified Eagle’s medium (Wako Pure Chemical Industries), supplemented with 10% fetal calf serum and penicillin/streptomycin.

### Constructs

Edit-R inducible Lentiviral Cas9 vector was obtained from GE Healthcare. Oligonucleotides encoding sgRNA against human centromere (29) or control, shown in Supplementary Table S2, were annealed, and then inserted into the pLKO.1-puro U6 sgRNA BfuAI stuffer lentivirus vector, (Addgene, #50920). The cDNAs for human RAD51 and RAD52 were amplified from reverse transcription products obtained from HT1080 cells by PCR using primers shown in Supplementary Table S2 and cloned into a pcDNA3 vector (Invitrogen) with a myc-tag at the N-terminus. All vectors used were verified by DNA sequencing.

### Virus production and transduction

Lentivirus was produced by transient transfection of Lenti-X 293T cells using polyethyleneimine ‘MAX’ transfection reagent (Polysciences, 24765) as described previously (32). Viral constructs were cotransfected with pMDL, pRev and pVSVG vectors (kind gifts from Dr Kinichi Nakashima, Kyushu University, Japan). Culture supernatants containing lentivirus were collected 48 h after transfection and cleared through a 0.45 μm cellulose acetate membrane filter. HT1080 and U2OS cells were transduced with virus-containing medium supplemented with 8 μg/ml polybrene by centrifugation at 1000 x g for 2 h at 32°C and then further cultured for 8 h. The virus-containing medium was removed and replaced with fresh medium.

### Isolation of stable expression clones

HT1080 and U2OS cells were transduced with lentivirus for Dox–Cas9 nuclease. At 48 h after virus transduction, cell culture medium was switched with the medium supplemented with 5 μg/ml blasticidin, and cells were further cultured for 1 week. Then, single clones stably expressing Dox–Cas9 nuclease were isolated by limited dilution methods. HT1080-Dox–Cas9 and U2OS-Dox–Cas9 cells were further transduced with lentivirus for sgControl or sgCentromere, and then virus-transduced cells were selected by 2 μg/ml puromycin for 1 week.

### Treatments of cells

To induce expression of Dox–Cas9 nuclease, cells were cultured for 24 h in medium supplemented with 200 ng/ml Dox (Fujifilm, 049–31121), unless otherwise noted.

To examine the time course of centromeric DSB repair, after treatment of cells with Dox for 24 h, the Dox-containing medium was removed, and the cells were washed thrice with phosphate-buffered saline (PBS) and cultured in fresh medium without Dox. The medium was changed daily. At indicated timepoints, cells were fixed with ice-cold methanol and kept at 4°C until performing immunofluorescence analysis.

For ionizing radiation, cells were radiated with 10 Gy using M-150WE X-ray generator (SOFTEX). After X-ray irradiation, cells were fixed with ice-cold methanol at indicated timepoints and kept at 4°C until performing immunofluorescence analysis. The cell culture medium was changed daily.

To arrest cells in the G2 and G1 phases of the cell cycle, RO-3306 (Sigma–Aldrich, SML0569) and Palbociclib (Selleck, S1116) were added into medium 12 h before Dox treatment at final concentrations of 10 and 1 μM, respectively.

For double-thymidine block (DTB) treatment, cells were first blocked with 2 mM thymidine (Sigma–Aldrich, 89270) for 15 h, released for 9 h and then treated with 2 mM thymidine with or without 200 ng/ml Dox for 18 h for second block and Dox–Cas9 induction.

For Edu incorporation analysis, cells treated with DTB were incubated with Edu in the presence of thymidine for 2 h. Incorporated Edu was labeled using Click-iT EdU Alexa Fluor 488 Imaging Kit (Thermo Fisher Scientific, C10337), according to manufacturer's protocol.

### Immunofluorescence analysis

**Figure 1.**
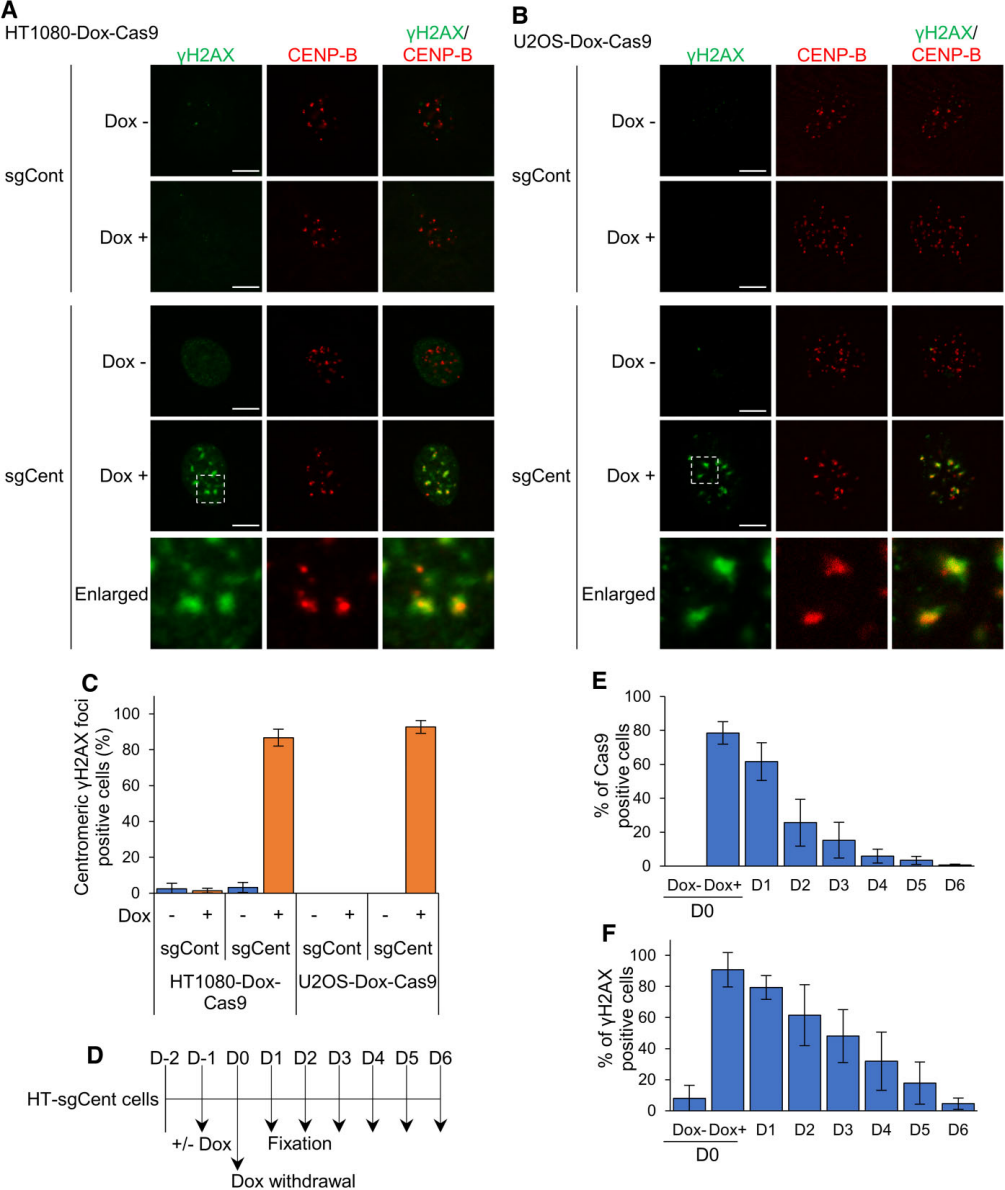
Establishment of an experimental model to elucidate the molecular mechanisms of centromeric DSB repair in cultured human cells. (**A, B**) Immunofluorescence images of γH2AX and CENP-B in HT1080 (**A**) and U2OS (**B**) cells stably expressing Dox–Cas9 nuclease and sgRNA for control (sgCont) or centromere (sgCent). Enlarged images of the region enclosed by a white dotted line are shown. Data are representative of three independent experiments. Scale bar, 5 μm. (**C**) Percentage of cells containing centromeric γH2AX foci. (**D**) Experimental scheme for the time course of centromeric DSB repair after Dox withdrawal. (**E, F**) Percentage of cells expressing Cas9 nuclease (**E**) and γH2AX-positive cells (**F**), determined by immunofluorescence analysis of cells treated as shown in (**D**). (**C, E, F**) Data represent the mean ± standard deviation (SD) of three independent experiments.

Immunofluorescence analysis was performed as described previously with minor modifications (33,34). Cells were cultured on 12-mm diameter glass coverslips placed in 24-well plates and pre-extracted in 0.1% Triton X-100 in PBS for 20 s prior to fixation. After quick wash with PBS, the cells were fixed with 4% paraformaldehyde in PBS for 15 min at room temperature. For immunofluorescence analysis using anti-γH2AX or anti-Cas9 antibodies alone (for Figure 1E and F, and Supplementary Figure S1B) and using anti-cyclin A2 rabbit polyclonal antibody (for Figure 5D and F), cells were fixed with ice-cold 100% methanol for 20 min at −20°C without pre-extraction. After fixation, cells were washed thrice with PBS, permeabilized/blocked with 1% non-fat dry milk in PBS containing 0.3% Triton X-100 for 30 min at room temperature, and subsequently incubated with primary antibodies diluted in 1% bovine serum albumin (BSA) in PBS containing 0.3% Triton X-100 overnight at 4°C. The antibodies used for immunostaining are listed in Supplementary Table S1. Following incubation with primary antibodies, the cells were washed thrice with PBS, and subsequently incubated with secondary antibodies conjugated with fluorescent dyes diluted in 1% non-fat dry milk in PBS containing 0.3% Triton X-100 for 1 h at room temperature. The cells were washed thrice with PBS, stained with 4',6-diamidino-2-phenylindole (DAPI), mounted using Fluorescence Mounting Medium (Dako) and examined using a TCS SP5 laser-scanning confocal microscope with 63×/1.4 NA oil objective lens (Leica Microsystems). Images were analyzed using the Image J software (National Institute of Health).

### Immunoblotting analysis

Immunoblotting analysis was performed as described previously with minor modifications (35–37). Whole cell lysates were prepared by incubating cells in the radio immunoprecipitation assay (RIPA) buffer (50 mM Tris-HCl, pH 7.5, 150 mM NaCl, 0.5% sodium deoxycholate, 0.1% sodiumdodecyl sulphate (SDS) and 1% Triton X-100) supplemented with complete ethylenediaminetetraaceticacid-free protease inhibitors (Roche) for 30 min at 4°C. Cell pellets were removed by centrifugation, and then the supernatants were mixed with Laemmli sample buffer after determination of protein concentration. Equal amounts of protein were resolved via SDS–polyacrylamide gelelectrophoresis and transferred to a nitrocellulose membrane (GE Healthcare). The membranes were blocked in TBST (10 mM Tris-HCl, pH 8.0, 150 mM NaCl and 0.1% Tween 20) containing 5% non-fat dry milk and then incubated overnight at 4°C with primary antibodies diluted in TBST containing 1% BSA. The antibodies used for immunoblotting are listed in Supplementary Table S1. Horseradish peroxidase-conjugated secondary antibodies (Jackson ImmunoResearch) and SuperSignal West Pico Chemiluminescent Substrate (Thermo Scientific) were used for the detection.

### Cell cycle analysis

DNA contents were determined by flow cytometry following cell fixation with 70% cold ethanol (20 min, on ice), 100 μg/ml RNase A treatment (15 min, 37°C) and 50 μg/ml propidium-iodide staining (15 min, room temperature). Stained cells were analyzed by FACSAria II (BD Biosciences), and data were analyzed using the FlowJo software (Tomy Digital Biology) as described previously (38,39).

### DNA and siRNA transfections, and WST-8 and apoptotic cell death assays

To express myc-tagged RAD51 and RAD52, cell culture medium was switched with the medium supplemented with 200 ng/ml Dox and then cells were transfected with the expression vectors using Lipofectamine 3000. The medium containing plasmid DNA was removed 8 h after transfection and replaced with fresh medium supplemented with Dox.

siRNAs were purchased from Thermo Scientific. Sequences for siRNA are shown in Supplementary Table S2. To examine the effects of siRNAs on the protein expression levels (Figure 7B), cells were seeded in six-well plates at a density of 3 × 10^5^ cells per well in 2 ml culture medium with siRNA (20 pmol)–lipofectamine RNAiMAX (5 μl, Invitrogen) complexes. The cells were harvested 48 h after transfection and analyzed by immunoblotting analysis as described above. To examine the effect of siRNAs on cell proliferation after centromeric DSB induction (Figure 7C–E), cells were seeded in 96-well plates at a density of 8 × 10^3^ cells per well in 100 μl culture medium with siRNA (1.25 pmol)–lipofectamine RNAiMAX (0.3 μl). To examine the effects of siRNAs on apoptotic cell death (Figure 7F and G), cells were seeded in six-well plates at a density of 1.2 × 10^5^ cells per well in 2 ml culture medium with siRNA (20 pmol)–Lipofectamine RNAiMAX (5 μl, Invitrogen) complexes; 24 h after transfection, cell culture medium was switched with the medium supplemented with 200 ng/ml Dox, and cells were cultured for 24 h. Then, the Dox-containing medium was removed, and cells were washed thrice with PBS and cultured in fresh medium without Dox. The medium was changed daily. At indicated timepoints, cellular proliferation was analyzed using Cell Counting Kit-8 (Dojindo) according to the manufacturer’s protocol. Apoptotic cell death was assessed on day 3 after Dox withdrawal by flow cytometry analysis using an APC Annexin V Apoptosis Detection kit (BD Biosciences) according to the manufacturer’s protocol.

### Statistical analysis

The data were expressed as the mean ± standard deviation. The statistical analyses were performed using an unpaired two-tailed Student’s t-test. A *P* < 0.05 denoted statistically significant difference.

## Results

### Establishment of an experimental model in cultured human cells to elucidate the molecular mechanisms of centromeric DSB repair

To investigate how DSBs that occur at centromeres are repaired, we modified the CRISPR-Cas9 system in cultured human cells, which were previously reported by Yilmaz *et al.*, where DSBs were specifically induced at centromeres (29). We used HT1080 cells, a human fibrosarcoma cell line, and U2OS cells, a human osteosarcoma cell line, both of which are widely used in many studies for DSB repair and centromere function (29,40,41). Dox–Cas9 nuclease and centromere-specific sgRNA were stably expressed in these cell lines. In the established cell lines, the foci formation of the DSB marker γH2AX was examined through immunofluorescence analysis using specific antibodies. The position of centromere was identified by detecting foci of the centromere specific proteins CENP-A and CENP-B. In both HT1080-Dox–Cas9 and U2OS-Dox–Cas9 cells, only upon Dox treatments, the foci formation of γH2AX at centromeres was observed in about 90% of cells expressing sgRNA for centromere (sgCent) (Figure 1A–C). In contrast, centromeric γH2AX foci were not formed in cells expressing sgRNA for control (sgCont) in the presence or absence of Dox (Figure 1A–C). These results indicate that DSBs are induced at centromeres in HT1080 and U2OS cells by expressing both Dox–Cas9 and sgCent.

To determine whether Dox–Cas9-mediated centromeric DSBs are repaired, we next examined the time course of disappearances of both Cas9 expression and γH2AX signals after Dox withdrawal in HT1080-Dox–Cas9-sgCent (HT-sgCent) cells (Figure 1D). The proportion of Cas9-expressing cells was promptly decreased after Dox withdrawal (Figure 1E). A decrease in γH2AX signals was similarly observed after Dox withdrawal, but it progressed more slowly than the decrease in the Cas9 expression levels (Figure 1F). Furthermore, the repair of Dox–Cas9-mediated centromeric DSBs showed a time course similar to the repair of DSBs induced by ionizing radiation (Supplementary Figure S1A and B). Taken together, these results indicate that we established an experimental model in which the expression of both sgCent and Cas9 nuclease results in DSBs at centromeres in cultured human cells, and the generated centromeric DSBs are properly repaired.

### Three pathways of NHEJ, HR and SSA contribute to centromeric DSB repair

**Figure 2.**
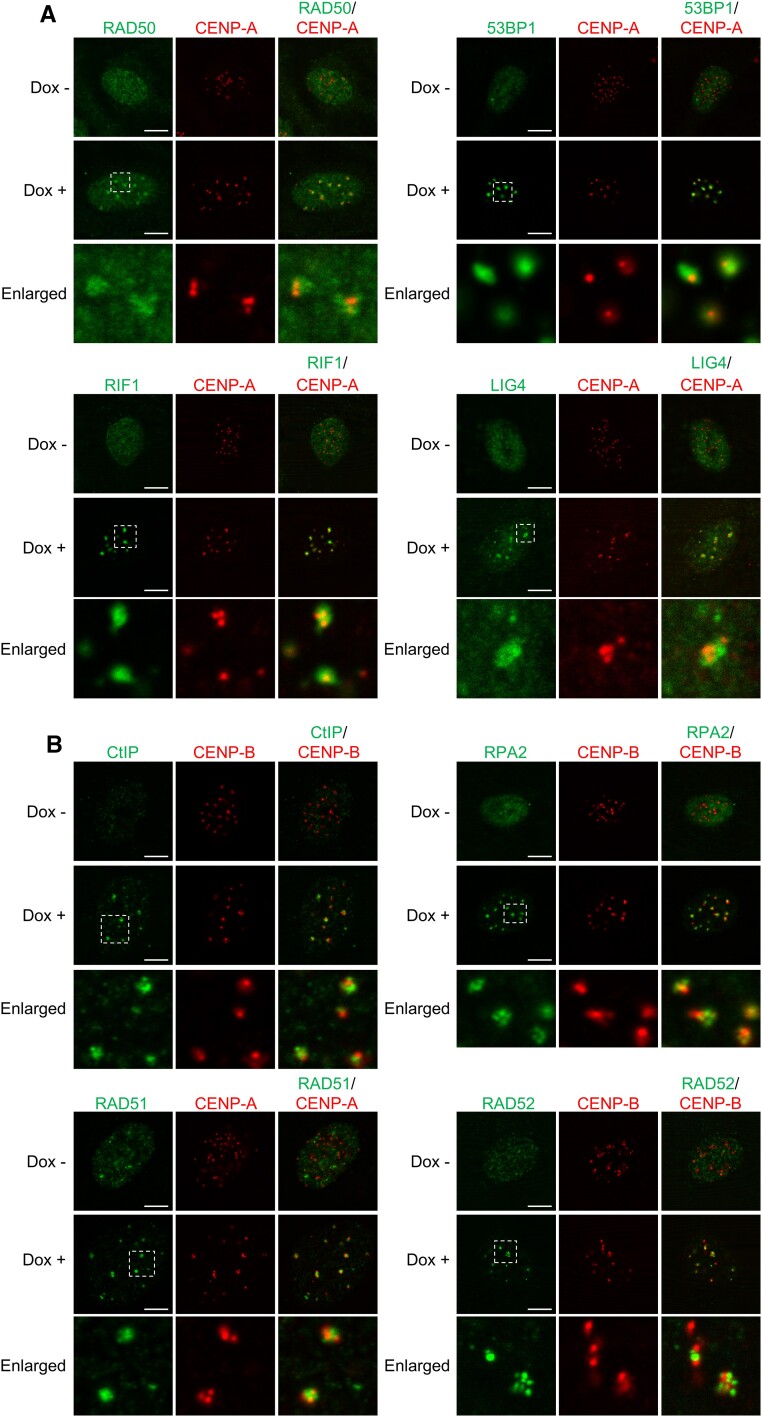
Cas9-induced centromeric DSBs recruit various repair factors involved in NHEJ, HR and SSA. (**A, B**) Immunofluorescence images of indicated repair factors and CENP-A/B in HT-sgCent cells treated (Dox +) or untreated (Dox -) with Dox. Enlarged images of the region enclosed by a white dotted line are shown. Data are representative of three independent experiments. Scale bar, 5 μm.

DSBs are mainly repaired by NHEJ and HR (10). In addition, SSA is considered to be an alternative repair pathway for DSBs at genomic regions bearing repetitive DNA sequences (26,27). However, it is unclear which pathways play predominant roles in DSB repair at centromeres. These repair pathways are defined by their own specific factors. 53BP1, RIF1 and LIG4 are involved in NHEJ, whereas CtIP and RPA2 function in both HR and SSA (10). On the other hand, RAD51 and RAD52 play particular roles in HR and SSA, respectively (10). Using the established cell lines expressing Dox–Cas9 and sgCent, we investigated the recruitments of various DSB repair factors involved in NHEJ, HR and SSA to centromeric DSB sites. In HT-sgCent cells, these repair factors formed foci that were colocalized with CENP-A/B foci upon Dox treatments (Figure 2A and B). The foci of these repair factors were colocalized with γH2AX foci (Supplementary Figure S2). Furthermore, foci formation of these repair factors at centromeres was also observed in U2OS-sgCent cells only upon Dox treatments (Supplementary Figure S3A and B). These results indicate that various repair factors involved in NHEJ, HR and SSA are recruited to centromeric DSB sites, suggesting that these three pathways contribute to centromeric DSB repair.

**Figure 3.**
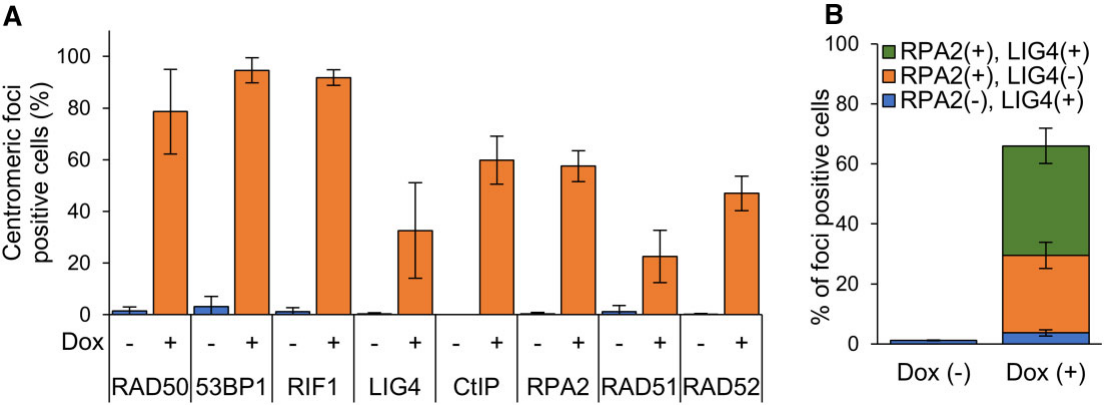
HR and SSA are employed more frequently for centromeric DSB repair than NHEJ. (**A**) Percentage of HT-sgCent cells containing centromeric foci of indicated repair factors. (**B**) Percentage of HT-sgCent cells containing foci of LIG4 and RPA2 concomitantly or individually, treated or untreated with Dox. (**A,B**) Data represent the mean ± SD of three independent experiments.

For several repair factors examined, there were differences in the proportions of cells with centromeric foci between HT-sgCent and U2OS-sgCent cells (Figure 3A and Supplementary Figure S3B). Especially, the proportions of cells with centromeric foci of LIG4 or RAD51 were lower in U2OS-sgCent cells compared to HT-sgCent cells. Therefore, HT-sgCent cells are considered to be more suitable for an experimental model to elucidate the molecular mechanisms of centromeric DSB repair compared to U2OS-sgCent cells. Accordingly, subsequent studies were conducted using HT-sgCent cells.

### HR and SSA are employed more frequently for centromeric DSB repair than NHEJ

To determine the relative contributions of the three repair pathways of NHEJ, HR and SSA to centromeric DSB repair, we compared the proportion of cells with centromeric foci between repair factors. About 30% of the Dox-treated cells showed centromeric foci of the NHEJ factor LIG4, whereas CtIP and RPA2, which are involved in both HR and SSA, were recruited to centromeric DSB sites in 60% of cells (Figure 3A). These results suggest that HR/SSA are employed more frequently for centromeric DSB repair than NHEJ.

Next, to elucidate whether NHEJ and HR/SSA occur simultaneously or separately in one cell to repair centromeric DSBs, we investigated the proportion of cells, which contained foci of LIG4 and RPA2 concomitantly or individually, upon centromeric DSB induction. About 35% of cells harbored foci of both LIG4 and RPA2 upon centromeric DSB induction (Figure 3B), indicating that NHEJ and HR/SSA function simultaneously to repair centromeric DSBs in a part of cells. On the other hand, the proportion of cells containing only LIG4 foci without RPA2 foci were only 5%, whereas about 25% of cells contained only RPA2 foci without LIG4 foci (Figure 3B), indicating that a part of cells uses mainly HR/SSA to repair centromeric DSBs. These results suggest that HR/SSA are employed more frequently for centromeric DSB repair than NHEJ, although HR/SSA and NHEJ function simultaneously to repair centromeric DSBs in a part of cells.

### Centromeric DSBs are mainly repaired through SSA

**Figure 4.**
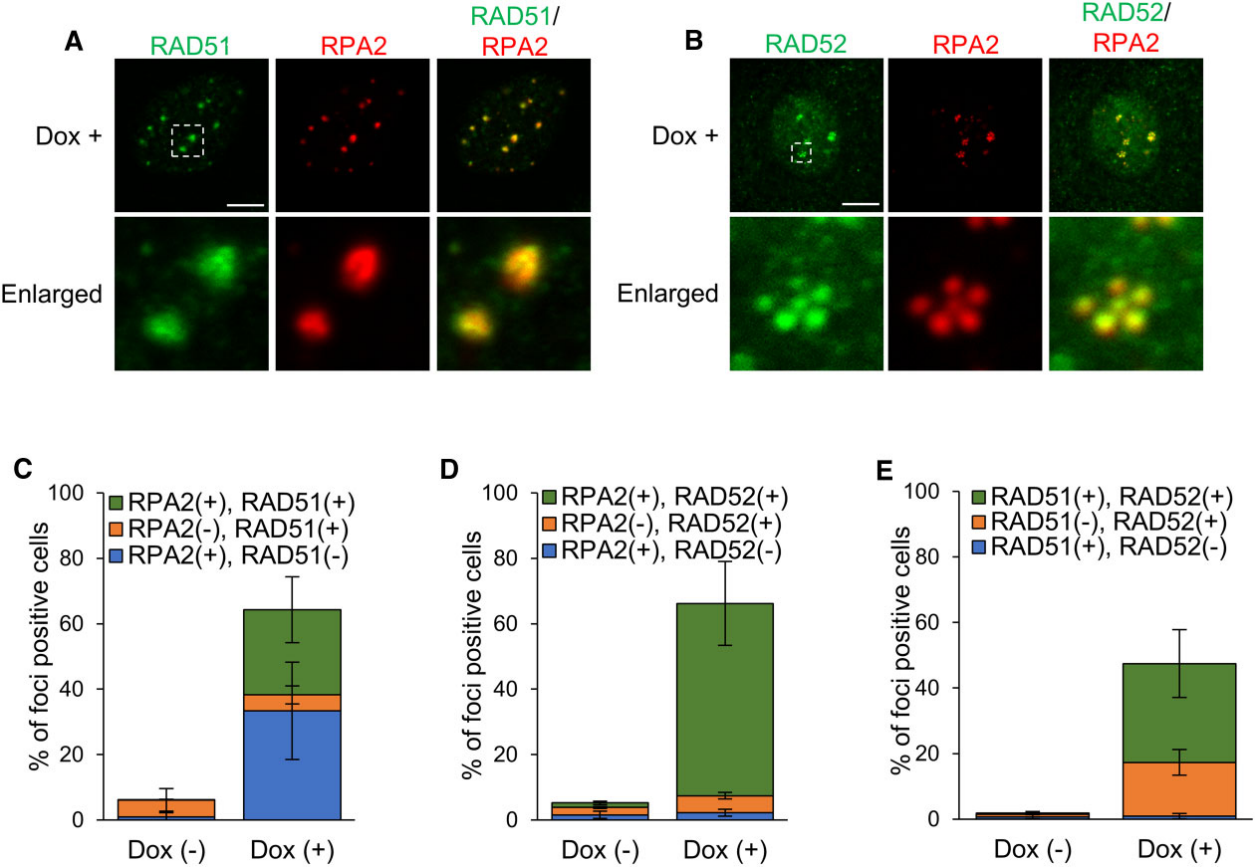
Centromeric DSBs are mainly repaired through SSA. (**A,B**) Immunofluorescence images of RPA2 and RAD51 (**A**), and RPA2 and RAD52 (**B**) in HT-sgCent cells treated with Dox. Enlarged images of the region enclosed by a white dotted line are shown. Data are representative of three independent experiments. Scale bar, 5 μm. (**C,D,E**) Percentage of HT-sgCent cells containing foci of RPA2 and RAD51 (**C**), RPA2 and RAD52 (**D**), and RAD51 and RAD52 (**E**) concomitantly or individually, treated or untreated with Dox. Data represent the mean ± SD of three independent experiments.

RPA is a DSB repair factor common to HR and SSA pathways (16,26). On the other hand, a factor that is involved in the replacement of RPA is different for HR and SSA. Downstream of RPA, RAD51 plays roles in HR, whereas RAD52 functions in SSA (10). The mechanisms that select RAD51 or RAD52 downstream of RPA for centromeric DSB repair are unknown. We investigated the spatial relationship in foci formation between RPA2 and RAD51 or RAD52 upon centromeric DSB induction. In cells containing both RPA2 and RAD51 or RAD52 foci simultaneously, centromeric RPA2 foci were completely colocalized with foci of either RAD51 or RAD52 (Figure 4A and B), which is consistent with that both RAD51 and RAD52 function downstream of RPA.

We further investigated the proportion of cells, which contained foci of RPA2 and RAD51 or RAD52 concomitantly or individually, upon centromeric DSB induction. While about 25% of cells were RPA2 and RAD51 foci double-positive cells, over 30% of cells contained centromeric foci of only RPA2, but not RAD51 (Figure 4C). In contrast, the majority of cells with centromeric RPA2 foci contained RAD52 foci upon centromeric DSB induction, and single-positive cells of each factor were hardly observed (Figure 4D). These results suggest that RAD52, rather than RAD51, cooperates with RPA2 to repair DSBs at the centromeres.

We next investigated the relationship in foci formation between RAD51 and RAD52 upon centromeric DSB induction. About 30% of cells contained centromeric DSB foci of both RAD51 and RAD52 (Figure 4E). On the other hand, about 15% of cells were RAD52 foci single-positive cells, whereas the cells with only RAD51 foci were hardly observed (Figure 4E). These results suggest that RAD52 predominates over RAD51 in centromeric DSB repair, although RAD51 and RAD52 functions simultaneously in a part of cells. Taken together, these results indicate that SSA plays major roles in centromeric DSB repair, although DSBs at centromeres are simultaneously repaired through NHEJ, HR and SSA in a part of cells.

### NHEJ acts on centromeric DSB repair throughout the cell cycle, whereas HR and SSA function in centromeric DSB repair in the S and G2 phases

**Figure 5.**
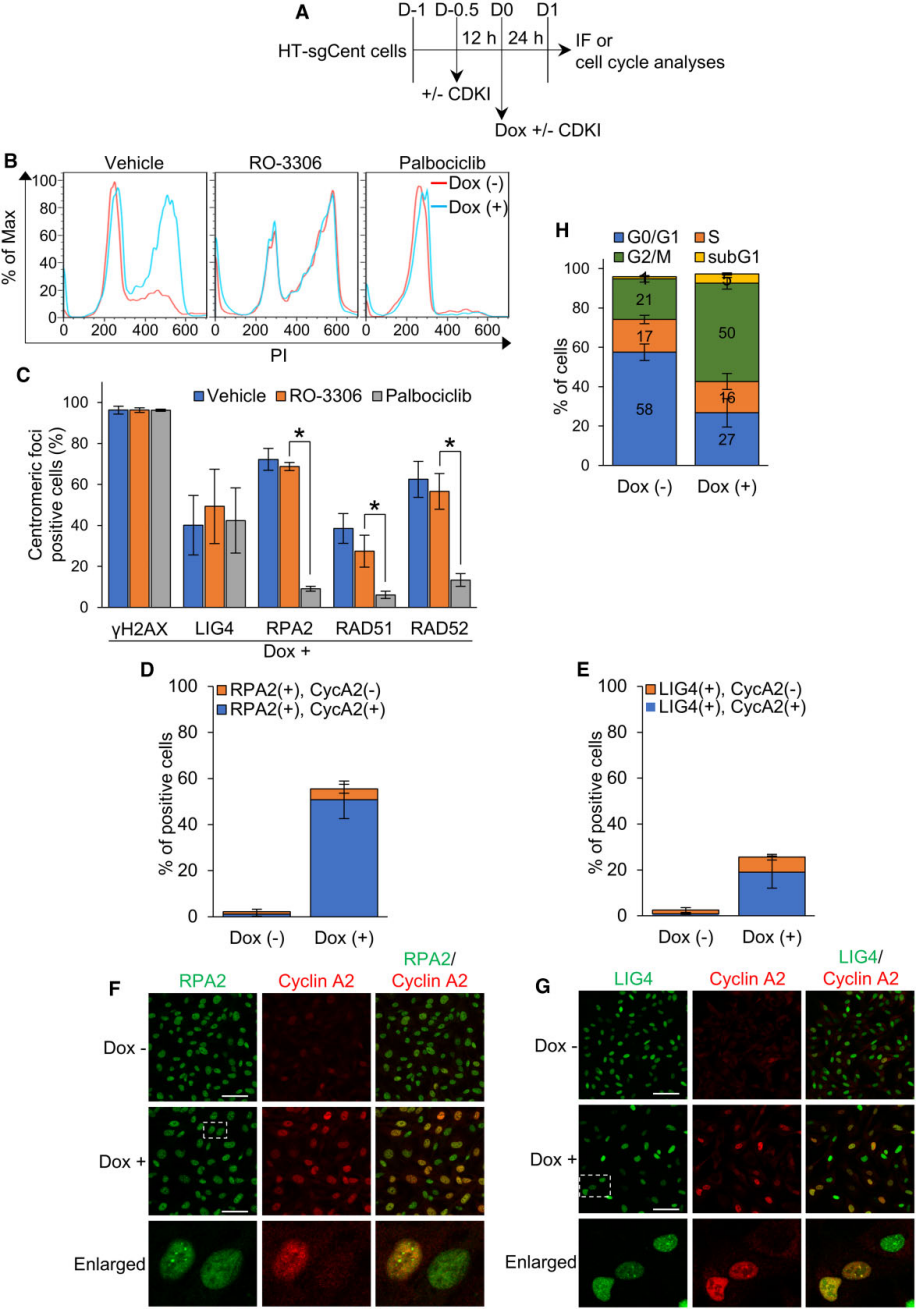
NHEJ acts on centromeric DSB repair throughout the cell cycle, whereas HR/SSA function in centromeric DSB repair in the S and G2 phases. (**A**) Experimental scheme to examine the effects of CDKI on the centromeric foci formation of repair factors and the cell cycle upon induction of centromeric DSBs. (**B**) DNA content analysis by flow cytometry in HT-sgCent cells treated as shown in (**A**), stained with propidium iodide. (**C**) Percentage of HT-sgCent cells containing centromeric foci of indicated repair factors, treated as shown in (**A**). (**D, E**) Percentage of HT-sgCent cells containing RPA2 (**D**) and LIG4 (**E**) foci with or without cyclin A2 (CycA2) expression. (**F, G**) Immunofluorescence images of CycA2, and RPA2 (**F**) or LIG4 (**G**) in HT-sgCent cells. Enlarged images of the region enclosed by a white dotted line are shown. Scale bar, 50 μm. (**H**) Percentage of each cell cycle population in HT-sgCent cells treated or untreated with Dox. (**C, D, E, H**) Data represent the mean ± SD of three independent experiments. *P < 0.05. (**B, F,G**) Data are representative of three independent experiments.

The cell cycle is a major determinant of DSB repair pathway choice (19–21). In general, NHEJ operates in all phases of the cell cycle, whereas HR occurs during the S and G2 phases. To investigate the recruitments of repair factors to centromeric DSB sites in each cell cycle phase, we arrested the cell cycle of HT-sgCent cells in the G1 and G2 phases by pretreating with inhibitors for cyclin-dependent kinases (CDKIs) and then induced centromeric DSBs through Cas9 nuclease induced by Dox treatment (Figure 5A). The effects of CDKIs on the cell cycle and the recruitments of repair factors to centromeric DSB sites were examined through flow cytometry and immunofluorescence analyses, respectively. As expected, RO-3306, an inhibitor of CDK1, increased the population of cells in the G2 phase, whereas Palbociclib, an inhibitor of CDK4/6, arrested the cell cycle in the G1 phase (Figure 5B). Treatments with these CDKIs did not affect the proportion of cells with centromeric γH2AX foci upon Dox treatment (Figure 5C), indicating that centromeric DSBs are properly induced in the presence of these CDKIs. The proportion of cells with centromeric LIG4 foci upon Dox treatment was not affected by either RO-3306 or Palbociclib. In contrast, RO-3306 treatment did not affect the proportion of cells with centromeric foci of RPA2, RAD51 and RAD52, whereas Palbociclib significantly suppressed foci formation of these repair factors at the centromeres upon Dox treatments. These results indicate that NHEJ repair factors are recruited to centromeric DSB sites in both G1 and G2 phases, whereas the recruitments of HR and SSA factors occur in the G2 phase, but not in the G1 phase.

We next investigated the expression of cyclin A2, an S and a G2 phases cyclin, in cells with centromeric foci of repair factors upon Dox treatment. Over 90% of RPA2 foci-positive cells expressed cyclin A2 (Figure 5D and F), indicating that HR/SSA repair factors are recruited to centromeric DSB sites during the S and G2 phases. In contrast, about 25% of centromeric LIG4 foci-positive cells did not express cyclin A2 (Figure 5E and G), indicating that NHEJ repair factors are recruited to centromeric DSB sites not only in the S and G2 phases but also in the G1 phase. These results suggest that NHEJ acts on centromeric DSB repair throughout the cell cycle, whereas HR/SSA function in centromeric DSB repair in the S and G2 phases. Thus, recruitments of NHEJ, HR and SSA factors to centromeric DSB sites are separately regulated temporally during the cell cycle.

Through the cell cycle analysis, we found that Dox treatments of HT-sgCent cells increased the proportion of cells in the G2 phase (Figure 5B and H). On the other hand, centromeric γH2AX foci were induced to a similar extent in both G1 and G2 phases upon Dox treatments (Figure 5C). These results suggest that centromeric DSB repair in the G2 phase is a more time-consuming process than the repair in the G1 phase. It was previously reported that NHEJ was a more efficient and rapid repair pathway than HR (42,43). Accordingly, these results suggest that centromeric DSBs occurred in the G2 phase are mainly repaired through HR/SSA, whereas those in the G1 phase are repaired through NHEJ, leading to the increase in cell population in the G2 phase upon centromeric DSB induction.

It was previously reported by Yilmaz *et al.* that centromeric DSBs were repaired via HR throughout the cell cycle, even in the G1 phase (29). In contrast, we showed in this study that the recruitments of RAD51 and RPA2 to centromeric DSB sites were significantly inhibited in the G1 phase. Yilmaz *et al.* reported that RAD51, RPA and BRCA1 formed centromeric DSB foci in the G1 phase in cells treated with DTB (29). Therefore, we investigated the effects of DTB treatment on the recruitment of repair factors to centromeric DSB sites in HT-sgCent cells (Supplementary Figure S4A). DTB treatment did not significantly inhibit the formation of centromeric DSB foci of RPA2 and RAD51 (Supplementary Figure S4B). These results were consistent with the study by Yilmaz *et al.* (29). Furthermore, the centromeric foci formation of γH2AX, LIG4 and RAD52 upon Dox treatments was also not affected by DTB treatment (Supplementary Figure S4B). However, a flow cytometry analysis of DNA contents showed that the cell cycle of HT-sgCent cells was mainly arrested in the S phase by DTB treatment (Supplementary Figure S4C). We further examined the incorporation of Edu to detect DNA replication and the expression of cyclin A2 as the marker of S and G2 phases in DTB-treated cells upon centromeric DSB induction by immunofluorescence analysis (Supplementary Figure S4D and E). The majority of DTB-treated cells with centromeric foci of RPA2 or RAD51 expressed cyclin A2 (Supplementary Figure S4D), indicating that the cell cycle of those cells is during the S and G2 phases. In contrast, Edu-incorporated cells with centromeric foci of RPA2 or RAD51 were not observed at all (Supplementary Figure S4E), indicating that DNA replication in those cells was completely suppressed. These results indicate that the cell cycle of cells containing centromeric DSB foci of HR factors after DTB treatment is the S phase but not the G1 phase, even though DNA replication is blocked in those cells. Taken together, all these results indicate that centromeric DSBs occurred in the S and G2 phases are mainly repaired through HR and SSA, whereas those in the G1 phase are repaired through NHEJ.

### NHEJ functions at both active and inactive centromeres, whereas HR and SSA act on DSB repair at inactive centromeres

**Figure 6.**
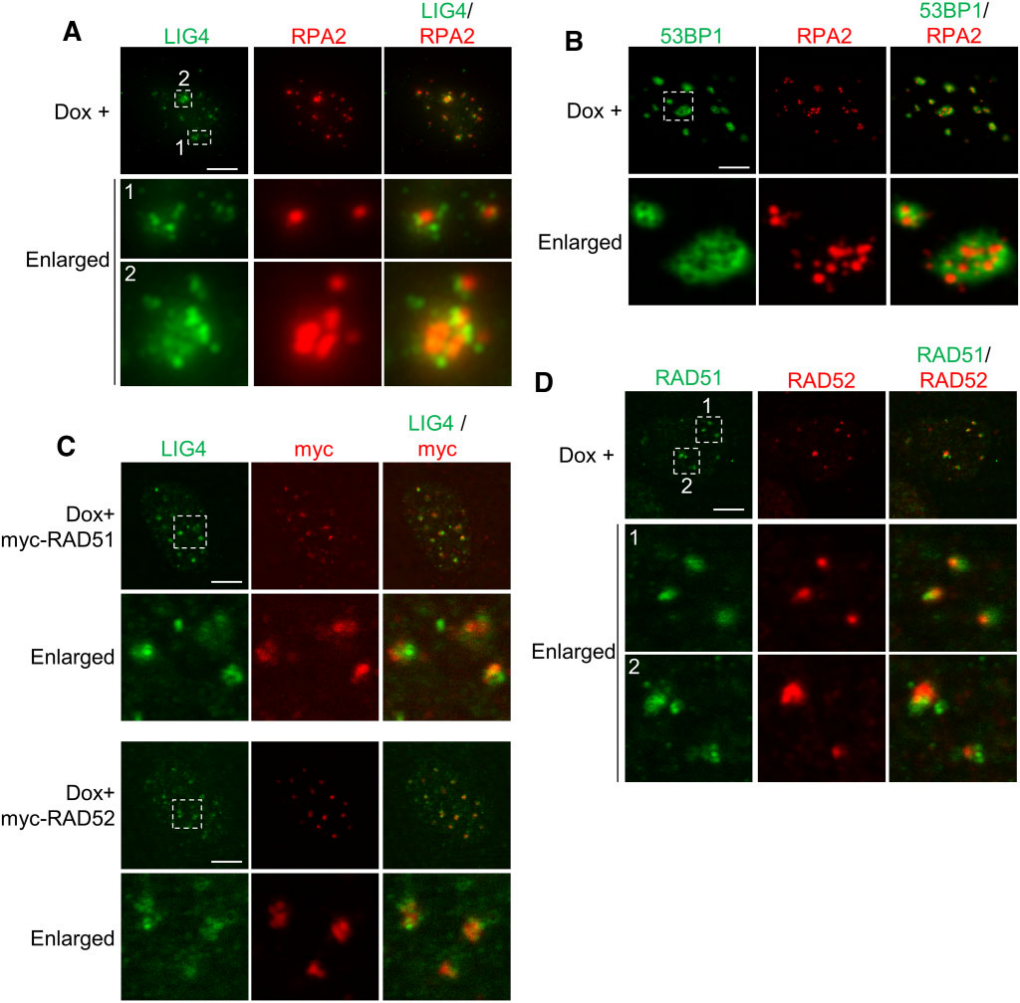
Recruitments of HR, SSA and NHEJ factors to centromeric DSB sites are spatially separately regulated. (**A, B**) Immunofluorescence images of RPA2, and LIG4 (**A**) or 53BP1 (**B**) in HT-sgCent cells treated with Dox. (**C**) Immunofluorescence images of LIG4, and myc-RAD51 or myc-RAD52 in HT-sgCent cells transfected with indicated expression vectors and treated with Dox. (**D**) Immunofluorescence images of RAD51 and RAD52 in HT-sgCent cells treated with Dox. (**A–D**) Enlarged images of the region enclosed by a white dotted line are shown. Data are representative of three independent experiments. Scale bar, 5 μm.

Next, we investigated in detail the spatial relationship of centromeric DSB foci between NHEJ and HR/SSA repair factors. As described above, upon centromeric DSB induction, about 35% of cells contained centromeric foci of both RPA2 and LIG4 simultaneously (Figure 3B). However, in cells containing both RPA2 and LIG4 foci, their foci were not colocalized despite being very closely located (Figure 6A). These results indicate that recruitments of NHEJ and HR/SSA factors to centromeric DSB sites are spatially separately regulated.

As shown in Figure 3A, 53BP1 was recruited to centromeric DSB sites in over 90% of cells. Thus, the majority of cells with centromeric RPA2 foci would contain 53BP1 foci. However, 53BP1 is known to inhibit the HR pathway upstream of RPA (19,20). Therefore, we investigated in detail the spatial relationship of centromeric DSB foci between 53BP1 and RPA2. Upon centromeric DSB induction, 53BP1 formed relatively large foci with 53BP1-negative regions (Figure 6B). Interestingly, RPA2 foci were only formed in such 53BP1-negative regions upon centromeric DSB induction (Figure 6B). These results are consistent with the function of 53BP1 in promoting NHEJ by suppressing HR (19,20), suggesting that 53BP1 plays important roles in spatial regulation of the DSB repair pathway choice between NHEJ and HR/SSA at centromeres.

To distinguish between HR and SSA in their spatial relationship with NHEJ, we attempted to perform immunofluorescence analysis of co-staining of LIG4 and RAD51 or RAD52. However, antibodies against endogenous RAD51 and RAD52 suitable for immunofluorescence analysis for co-staining with LIG4 were not available. Thus, we transiently expressed myc-tagged RAD51 (myc-RAD51) and RAD52 (myc-RAD52) in HT-sgCent cells and examined their colocalization with endogenous LIG4 upon centromeric DSB induction through immunofluorescence analysis using anti-myc and anti-LIG4 antibodies. Centromeric DSB foci of LIG4 were not colocalized with foci of either myc-RAD51 or myc-RAD52 despite being very closely located (Figure 6C). These results indicate that recruitments of NHEJ factors to centromeric DSB sites are spatially regulated separately from HR and SSA factors. Thus, NHEJ functions in distinct regions from either HR or SSA to repair DSBs at centromeres.

Next, we investigated in detail the spatial relationship of centromeric DSB foci between HR and SSA repair factors. In cells with centromeric DSB foci of both RAD51 and RAD52, RAD51 and RAD52 foci were not colocalized despite being very closely located (Figure 6D). These results indicate that HR and SSA function in DSB repair at different regions of centromeres. Taken together, these results indicate that the recruitments of HR, SSA and NHEJ factors to centromeric DSB sites are spatially separately regulated.

Finally, by comparing enlarged images in Figure 2 again in detail, we found that localizations of repair factor foci relative to CENP-A/B foci were different between NHEJ and HR/SSA factors. Centromeric DSB foci of 53BP1, RIF1 and LIG4, which are involved in the NHEJ pathway, were mainly formed at CENP-A/B foci and their vicinities (Figure 2A). In contrast, CtIP, RPA2, RAD51 and RAD52, which are involved in the HR and/or SSA pathways, were predominantly recruited to the edges of CENP-A/B foci and their vicinities upon centromeric DSB induction (Figure 2B). These results indicate that NHEJ functions at both active and inactive centromeres, whereas HR and SSA act on centromeric DSB repair only at inactive centromeres.

### NHEJ and HR play crucial roles in appropriate centromeric DSB repair, whereas SSA-mediated repair of centromeric DSBs leads to subsequent cell death

**Figure 7.**
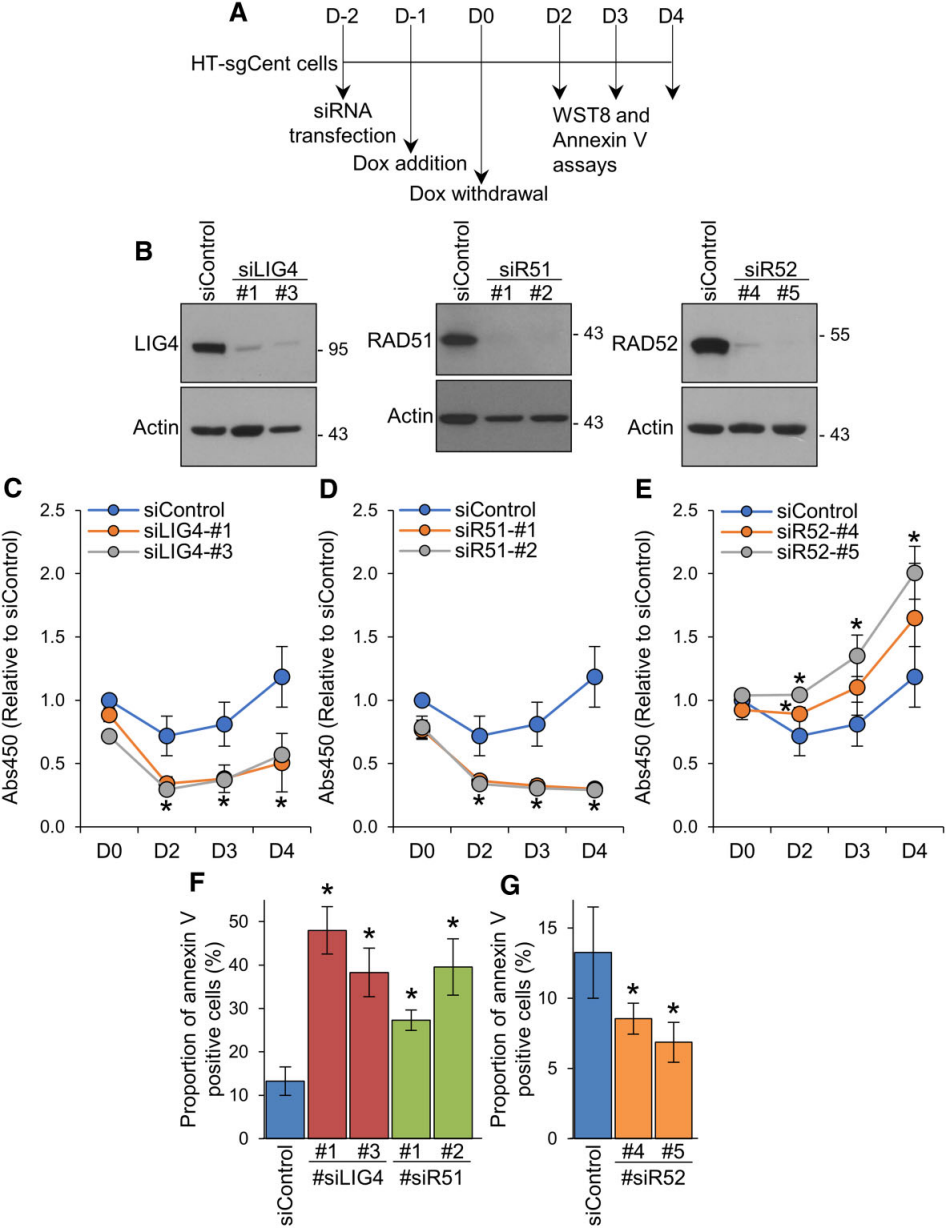
NHEJ and HR play crucial roles in appropriate centromeric DSB repair, whereas SSA-mediated repair of centromeric DSBs leads to subsequent apoptotic cell death. (**A**) Experimental scheme to examine the effects of siRNA-mediated knockdown of repair factors on cell proliferation and apoptotic cell death after Dox withdrawal. (**B**) Immunoblotting analysis of HT-sgCent cells transfected with indicated siRNAs. Cells were harvested 48 h after transfection and then analyzed. Actin was used as a loading control. (**C–E**) Cell proliferation curve of HT-sgCent cells treated as shown in (**A**), determined by WST-8 assay. (**F, G**) Percentages of apoptotic cells treated as shown in (**A**). Apoptotic cell death was assessed on day 3 after Dox withdrawal by annexin V staining followed by flow cytometry analysis. (**C–G**) Data represent the mean ± SD of three independent experiments. *P < 0.05, against siControl.

To elucidate the consequences of centromeric DSB repair through NHEJ, HR and SSA, we investigated the effects of depletion of repair factors on cell proliferation after centromeric DSB induction (Figure 7A). Expression of LIG4, RAD51 or RAD52 was knocked down using siRNAs specific for each target gene. To reduce the possibility of off-target effects by siRNA, two different siRNAs for each target were tested. The decrease in the expression levels of each protein was confirmed by immunoblotting analysis (Figure 7B). The time course of cell proliferation after centromeric DSB induction was examined using WST-8 assay (Figure 7A). In cells transfected with siControl, the cell number moderately decreased on day 2 after Dox withdrawal and then gradually increased on days 3 and 4 (Figure 7C–E). Depletion of either LIG4 or RAD51 resulted in a further reduction in the cell number on day 2 after Dox withdrawal compared to cells transfected with siControl (Figure 7C and D). Furthermore, in LIG4-depleted cells, although a slight increase in the cell number was observed, subsequent cell proliferation on days 3 and 4 was remarkably inhibited (Figure 7C). In contrast, an increase in the cell number on days 3 and 4 was entirely abolished by RAD51 depletion (Figure 7D). These results indicate that both LIG4 and RAD51 are required for repair of centromeric DSBs. Interestingly, in RAD52-depleted cells, a decrease in the cell number on day 2 after Dox withdrawal, which was observed in siControl-transfected cells, did not occur, followed by cell proliferation without any delay (Figure 7E). These results suggest that centromeric DSB repair through RAD52 has a detrimental effect on cell proliferation.

To elucidate the mechanism by which depletion of repair factors affects cell proliferation after centromeric DSB induction, we next investigated the effects of siRNA-mediated knockdown of repair factors on apoptotic cell death after centromeric DSB induction. The proportion of apoptotic cells was determined by annexin V staining followed by flow cytometry analysis. Depletion of either LIG4 or RAD51 markedly increased the proportions of apoptotic cells on day 3 after Dox withdrawal, compared to those in cells transfected with siControl (Figure 7F), indicating important roles of LIG4 and RAD51 in centromeric DSB repair. By contrast, in RAD52-depleted cells, the proportions of apoptotic cells on day 3 after Dox withdrawal were significantly decreased, compared to those in siControl-transfected cells (Figure 7G). These results, together, indicate that apoptotic cell death observed after centromeric DSB induction is predominantly attributed to RAD52-mediated repair of centromeric DSBs. Thus, these results suggest that NHEJ and HR play crucial roles in appropriate centromeric DSB repair, whereas SSA-mediated repair of centromeric DSBs leads to subsequent apoptotic cell death, at least in a certain proportion of cells.

## Discussion

Centromeres are chromosomal regions that are prone to be damaged, but the regulatory mechanisms of DSB repair at the centromeres remained unknown. In this study, we investigated the recruitments of various DSB repair factors to centromeric DSBs induced by CRISPR-Cas9 in cultured human cells, and demonstrated that the three pathways of NHEJ, HR and SSA were involved in the DSB repair at centromeres (Figures 2 and 3 and Supplementary Figures S2 and S3). Among these three pathways, SSA was most frequently employed for centromeric DSB repair (Figure 4). For centromeric DSB repair, NHEJ occurred throughout the cell cycle, whereas HR and SSA were active during the S and G2 phases (Figure 5), similar to the general repair of non-centromeric DSBs. We also showed that the repair factors involved in HR, SSA and NHEJ were recruited concomitantly to centromeric DSB sites, but their foci were not colocalized despite being very closely located (Figure 6). Furthermore, NHEJ factors are recruited to both active and inactive centromeres, whereas HR and SSA factors are recruited only to inactive centromeres (Figure 2). Importantly, SSA-mediated repair of DSBs at centromeres leads to subsequent apoptotic cell death, whereas HR and NHEJ are required for appropriate centromeric DSB repair (Figure 7), which may suggest that SSA-mediated DSB repair in inactive centromeres causes dysregulation of centromere function through error-prone DSB repair. These findings would lead to a better understanding of the molecular mechanisms underlying the development of genome instability and aneuploidy caused by dysregulation of the centromeres.

### Temporal regulation of centromeric DSB repair by the cell cycle

We here revealed that the choice of DSB repair pathway at centromeres were regulated by the cell cycle, similar to a general DSB repair at non-centromeric genome regions. To repair centromeric DSBs, NHEJ occurred throughout the cell cycle, whereas HR and SSA were mainly active during the S and G2 phases (Figure 6). On the other hand, Yilmaz *et al.* previously reported that centromeric DSBs were repaired by HR throughout the cell cycle, even in the G1 phase, where HR is believed not to be operational (29). In this study, we showed that Palbociclib, which is a CDK4/6 inhibitor, caused the cell cycle arrest in the G1 phase (Figure 6B) and inhibited the recruitments of HR repair factors of RAD51 and RPA2 to centromeric DSB sites (Figure 6C). On the other hand, Yilmaz *et al.* showed that RAD51, RPA and BRCA1 were recruited to centromeric DSB sites in cells treated with DTB, and concluded that these HR repair factors were recruited to centromeric DSB sites in the G1 phase (29). In general, however, thymidine is known to interfere with nucleotide metabolism, resulting in the depletion of nucleotides for DNA synthesis (44). Thus, DTB treatment is widely used to synchronize the cell cycle by arresting in the early S phase (45,46). These general knowledges are consistent with our results that DTB treatments in HT-sgCent cells arrested the cell cycle in the S phase, which was shown by immunofluorescence analysis for cyclin A2 and flow cytometry analysis of DNA contents (Supplementary Figure S4). On the other hand, Yilmaz *et al.* identified the cell cycle of DTB-treated cells, which were negative for both Edu-incorporation and a phosphorylated Ser10 at the histone H3, as the G1 phase. However, as shown in Supplementary Figure S4D and E, the majority of DTB-treated cells did not incorporate Edu, but expressed cyclin A2, indicating that the cell cycle in these cells was the S phase, even though DNA replication was inhibited. These results suggest that it is inappropriate to determine the cell cycle of DTB-treated cells based on their abilities to incorporate Edu. Therefore, the cell cycle of cells containing centromeric DSB foci of HR factors after DTB treatment is the S phase but not the G1 phase. We here conclude that centromeric DSBs generated in the G1 phase are mainly repaired through NHEJ, whereas the three pathways of NHEJ, HR and SSA contribute centromeric DSB repair during the S and G2 phases.

### Spatial regulation of centromeric DSB repair through HR, SSA and NHEJ

Human centromeres are defined by the presence of tandem repeats composed of α-satellite DNA, which are further arranged into HORs that expand for several megabases (3). CENP-A protein is only deposited into functionally active centromeric regions, where kinetochore complex binds, within the centromeres (4–6). Here, we provided the results that the repair factors of NHEJ, HR and SSA were separately recruited to the distinct regions of centromeres (Figures 2 and 6). NHEJ repair factors were recruited to the regions of CENP-A foci and their vicinities (Figure 2A), whereas HR and SSA factors were recruited to the edges of CENP-A foci and their vicinities (Figure 2B). Importantly, Tsouroula *et al.* previously reported using super-resolution microscopy that, when centromeric DSBs were induced by Cas9 nuclease in NIH 3T3 mouse fibroblast cells, RAD51 formed foci at the edges of centromeres but not at centromere itself (47), which is definitely consistent with our results in this study. Therefore, these results suggest that DSBs at the active centromeres are repaired mainly through NHEJ, whereas the three pathways of HR, SSA and NHEJ function in the repair of DSBs that occur at the inactive centromeres, in human and mouse cells.

It is known that, in addition to cell cycle, transcription and epigenetic status at DSB sites play crucial roles in repair pathway choice at non-centromeric genome regions (16–22). Epigenetic status of active and inactive centromeres are different; the active centromere is an euchromatic region containing a di-methylated Lys4 at the histone H3, whereas the inactive centromere is composed of a heterochromatin containing a tri-methylated Lys9 at the histone H3 (48,49). Furthermore, it is known that non-coding RNAs are transcribed from centromeres, but its regulation is different in active and inactive centromeres (50–55). Therefore, these differences in transcriptional and epigenetic status at active and inactive centromeres would participate in the choice of repair pathways among HR, SSA and NHEJ. Repair of centromeric DSBs through HR and SSA during the S and G2 phases may lead to centromere fragility due to aberrant chromosome fusions and sister chromatid exchanges. To prevent the development of such centromere fragility, DSB repair through HR and SSA may be inhibited at the active centromeres, and instead NHEJ may mainly repair them.

### Consequences of centromeric DSB repair through HR, SSA and NHEJ

Centromeric DNA is challenged by various physiological processes, including DNA replication and non-coding RNA transcription, which lead to the generation of DSBs (56). Repair of DSBs at centromeres through error-prone repair pathways results in loss of α-satellite DNAs and gross chromosomal rearrangements, both of which drive tumorigenesis (30). However, it remained elusive which pathways were involved in DSB repair at centromeres. Here, for the first time to our knowledge, we showed that endogenous molecule of the SSA factor RAD52 was recruited to DSB sites at native centromeres in human cells. These results indicate that centromeric DSBs are repaired also by SSA, in addition to HR and NHEJ that have been previously reported. Furthermore, the relative contributions of the three pathways to centromeric DSB repair were unequal, with SSA-mediated repair being most frequently employed (Figure 4). Nevertheless, depletion of RAD52 inhibited apoptotic cell death which occurred after centromeric DSB induction (Figure 7E and G). These results indicate that SSA-mediated centromeric DSBs repair leads to subsequent apoptotic cell death, at least in a certain proportion of cells. Given that SSA is an error-prone repair pathway (26,27), the repair of DSB at inactive centromeres through SSA may cause centromere dysfunction, leading to cell death by chromosome instability.

In contrast, depletion of RAD51 markedly enhanced apoptotic cell death after centromeric DSB induction and completely blocked subsequent cell proliferation (Figure 7D and F). Consistently, it was previously shown that both pharmacological inhibition and siRNA-mediated depletion of RAD51 resulted in an increase in chromosomes with broken centromeres and translocations after centromeric DSB induction (29). Furthermore, it was previously reported in fission yeast that RAD51 suppressed gross chromosomal rearrangement at centromeres (57). These results together indicate that HR is an essential pathway for appropriate repair of DSBs at inactive centromeres and plays crucial roles in protection of integrity at the centromeres.

Tsouroula *et al.* reported previously in NIH 3T3 mouse fibroblast cells that the NHEJ repair factor KU80 was recruited to the centromeric DSB sites induced by Cas9 nuclease and sgRNA for minor satellite DNA, which constitutes the centromeres at mouse chromosomes in a small proportion of cells (5–10%) (47). In agreement with this finding, we observed that LIG4 formed centromeric DSB foci in a small proportion of HT1080 (30%) and U2OS (10%) cells (Figure 3A and Supplementary Figure S3B). Therefore, the proportion of cells, in which centromeric DSBs are mainly repaired through NHEJ, would not be markedly high. On the other hand, in this study, depletion of LIG4 remarkably promoted apoptotic cell death after centromeric DSB induction (Figure 7C and F). Furthermore, it was previously reported in human cells that DSBs induced by ionizing radiation at centromeres were repaired more efficiently rather than other genomic regions through NHEJ (28). These findings indicate that NHEJ plays important roles in the repair of DSBs at both active and inactive centromeres to prevent dysfunction of centromeres.

Taken together, although DSBs at centromeres are repaired through HR, SSA and NHEJ, SSA is most frequently used to repair centromeric DSBs. The temporal regulation of repair pathway choice for centromeric DSBs follows the same rules as for general non-centromeric DSB repair. Namely, NHEJ functions throughout the cell cycle, whereas HR and SSA occur during the S and G2 phases. While DSBs at active centromeres are repaired via NHEJ, the three pathways of NHEJ, HR and SSA are involved in DSB repair at inactive centromeres. Furthermore, NHEJ and HR play crucial roles in appropriate DSB repair at the centromeres, whereas SSA-mediated repair of DSBs at inactive centromeres leads to subsequent apoptotic cell death. Thus, DSB repair at inactive centromeres through SSA may cause the dysfunction of centromere through error-prone DSB repair.

## Supplementary Material

gkae852_Supplemental_File

## Data Availability

The data underlying this article are available in the article and in its online supplementary material.
