# Supplementary material for: RAD52-mediated repair of DNA double-stranded breaks at inactive centromeres leads to subsequent apoptotic cell death
Source: Nucleic Acids Res. 2024 Oct 3;52(21):12961–75. doi: 10.1093/nar/gkae852 (PMC11602138; doi:10.1093/nar/gkae852)
Supplement: gkae852_Supplemental_File [file gkae852_supplemental_file.pdf]

SUPPLEMENTARY MATERIALS

RAD52-mediated repair of DNA double-stranded breaks at inactive centromere leads to subsequent apoptotic cell death

Gen Maruta, Hisanori Maeoka, Toshiyuki Tsunoda, Kozaburo Akiyoshi, Satoshi Takagi, Senji Shirasawa and Shuhei Ishikura

Supplementary Figure legends

Supplementary Figures S1-S4

Supplementary Table S1

List of antibodies.

Supplementary Table S2

List of nucleotide sequences of primers and siRNAs.

Supplementary Figure legends

Supplementary Figure 1. Time course of DSB repair after ionizing radiation. (**A**) Experimental scheme for the time course of DSB repair after ionizing radiation (IR). (**B**) Percentage of γH2AX-positive cells, determined by immunofluorescence analysis of cells treated as shown in (A).

Supplementary Figure 2. Various repair factors involved in NHEJ, HR and SSA form foci colocalized with γH2AX foci upon induction of centromeric DSBs. Immunofluorescence images of indicated repair factors and γH2AX in HT-sgCent cells treated with Dox. Enlarged images of the region enclosed by a white dotted line are shown. Data are representative of three independent experiments. Scale bar, 5 μm.

Supplementary Figure 3. Recruitments of various repair factors to centromeric DSBs in U2OS cells. (**A**) Immunofluorescence images of indicated repair factors and CENP-A/B in U2OS-sgCent cells. Data are representative of three independent experiments. Scale bar, 5 μm. (**B**) Percentage of U2OS-sgCent cells containing centromeric foci of indicated repair factors. Data represent the mean ± SD of three independent experiments.

Supplementary Figure 4. Double-thymidine block treatment results in the cell cycle arrest in the S phase. (**A**) Experimental scheme to examine the effects of double-thymidine block (DTB) treatment on the foci formation of repair factors and the cell cycle upon induction of centromeric DSB. Asyn; asynchronized. (**B**) Percentage of HT-sgCent cells containing centromeric foci of indicated repair factors, determined by immunofluorescence analysis of cells treated as shown in (A). (**C**) DNA content analysis by flow cytometry in HT-sgCent cells treated as shown in (A), stained with propidium iodide (PI). Data are representative of three independent experiments. (**D**) Percentage of HT-sgCent cells containing RPA2 or RAD51 foci with or without cyclin A2 (CycA2) expression, determined by immunofluorescence analysis of cells treated as shown in (A). (**E**) Percentage of HT-sgCent cells containing RPA2 or RAD51 foci with or without Edu incorporation, determined by immunofluorescence analysis of cells treated as shown in (A). (**B,D,E**) Data represent the mean ± SD of three independent experiments.

Supplementary Figure 1

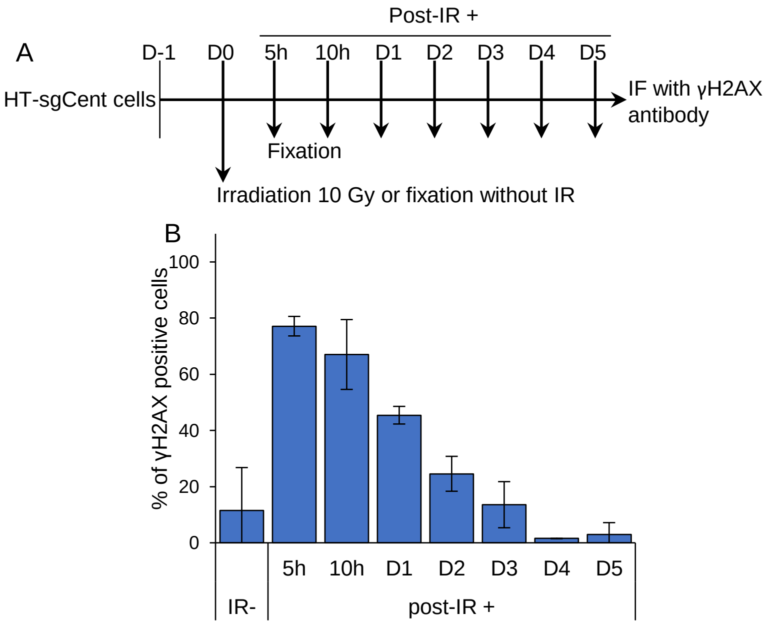

Supplementary Figure 2

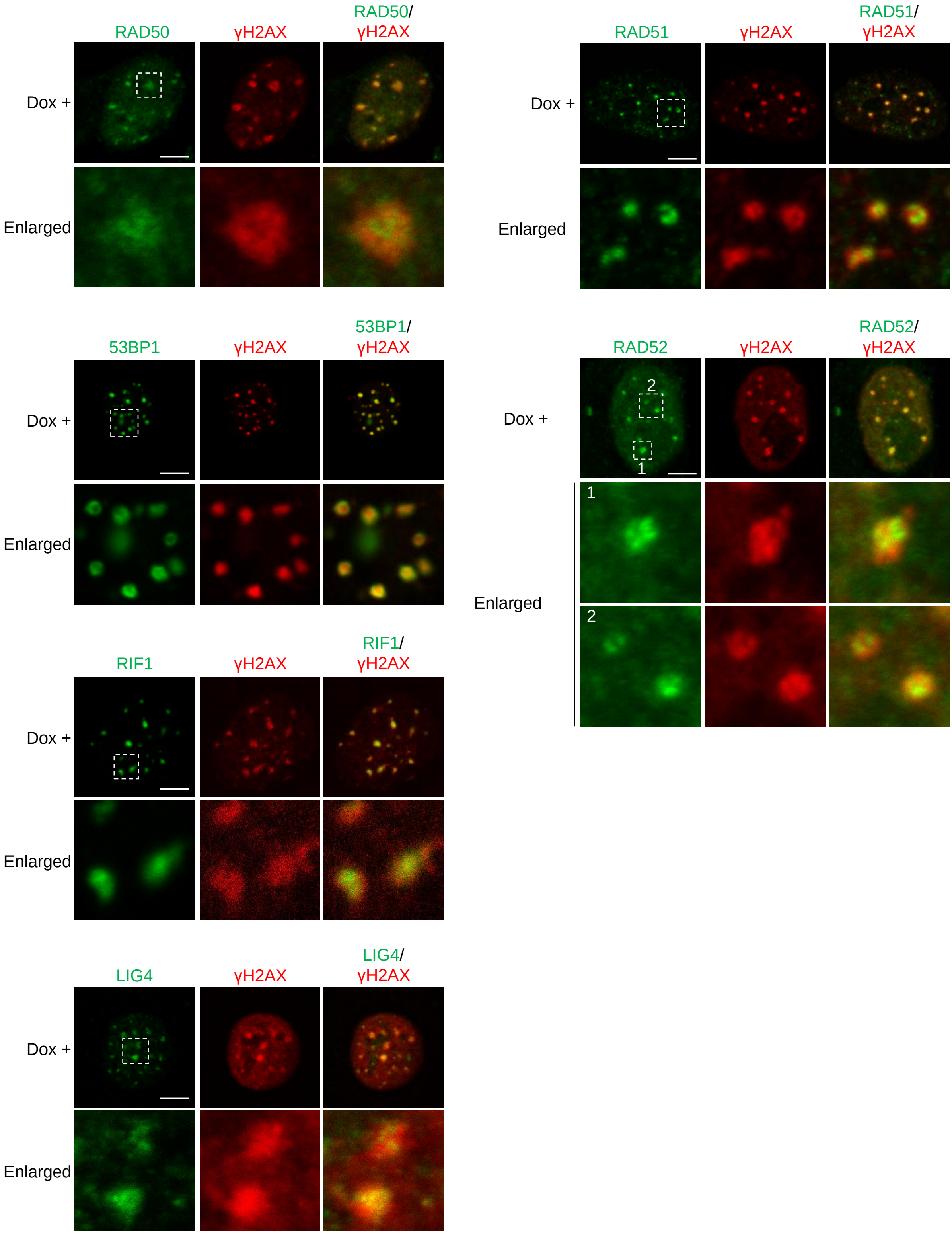

Supplementary Figure 3

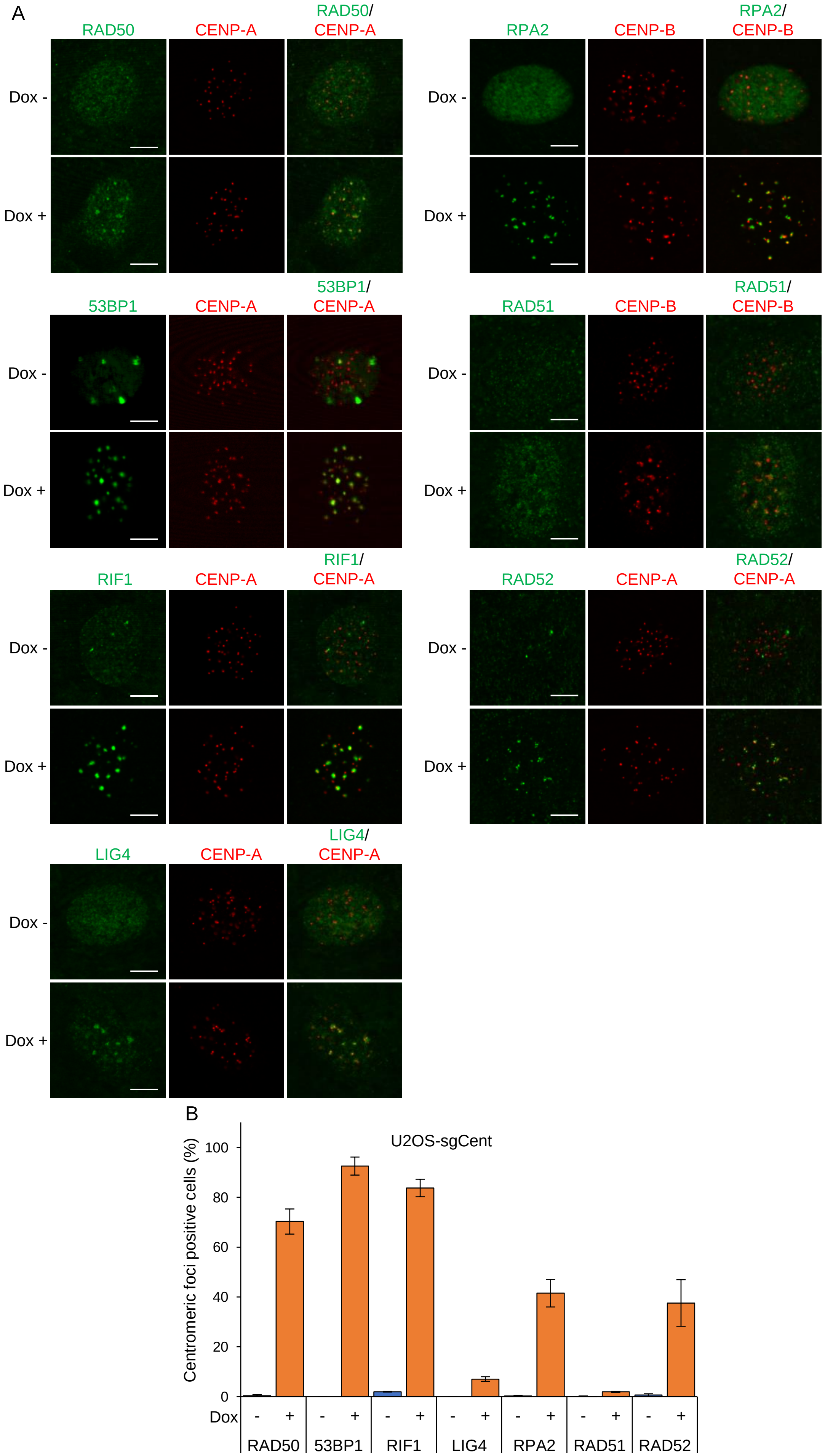

Supplementary Figure 4

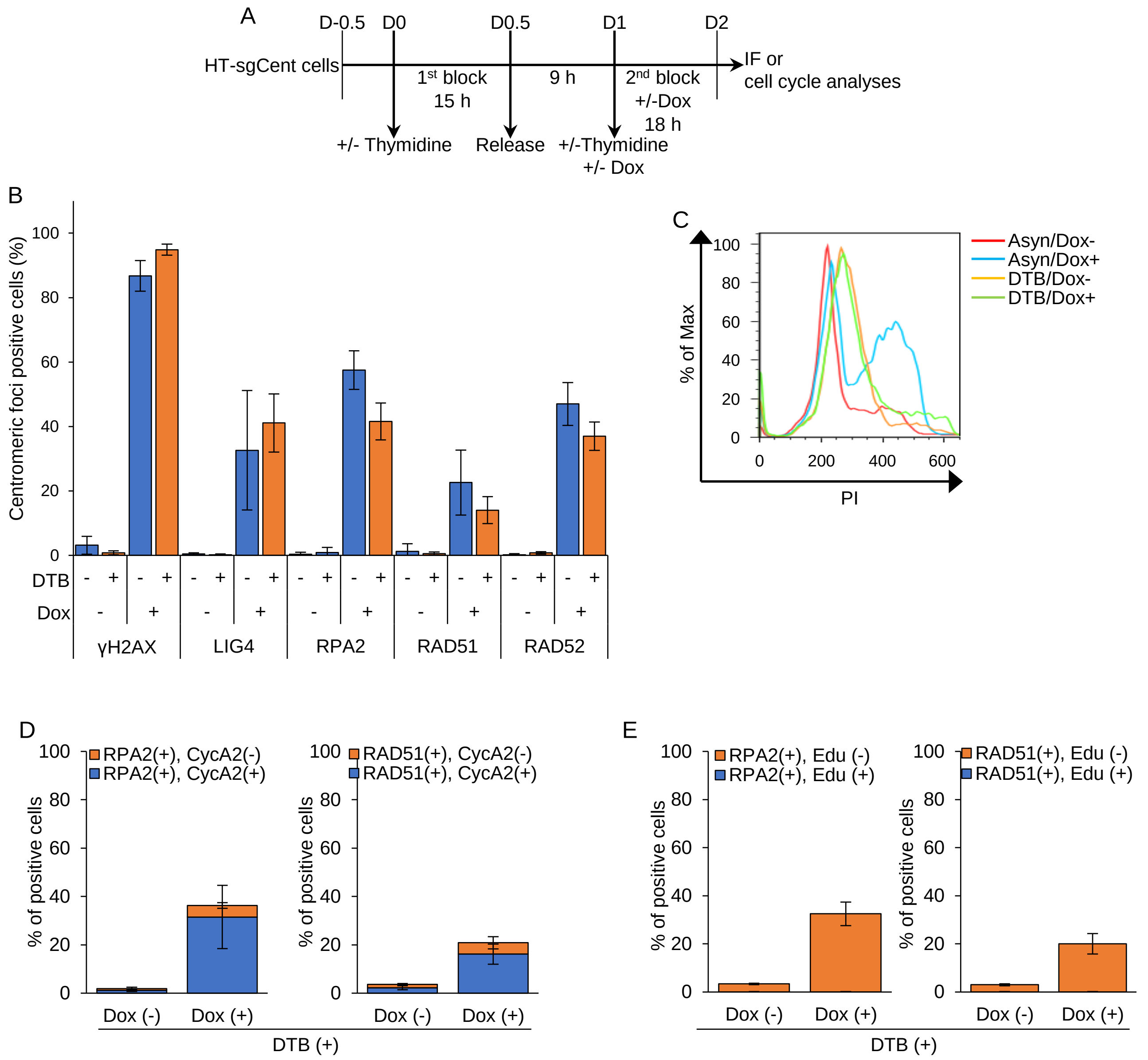

Supplementary Table S1

Primary antibodies	Species	Company	Catalog #	Dilution WB	Dilution ICC
CENP-A	Ms	GeneTex	GTX13939		1:500
CENP-B	Ms	SANTA CRUZ	sc-376392		1:500
CENP-B	Rb	Abcam	ab25734		1:1000
Cas9	Ms	SANTA CRUZ	sc-517386		1:300
γH2AX	Ms	Millipore	05-636		1:4000
RAD50	Rb	Abcam	ab124682		1:1000
53BP1	Rb	NOVUS	NB100-304		1:4000
RIF1	Rb	CST	95558		1:2000
DNA ligase IV (LIG4)	Rb	Abcam	ab193353	1:5000	1:2000
CtIP	Ms	SIGMA-ALDRICH	SAB2702168		1:500
RPA2	Rt	CST	2208		1:1000
RAD51	Rb	Abcam	ab133534	1:10000	1:1000
RAD51	Ms	Millipore	05-530-I-25UG		1:1000
RAD52	Rb	Abcam	ab124971	1:1000	1:1000
c-Myc	Ms	SANTA CRUZ	sc-40		1:500
Cyclin A2	Rb	SANTA CRUZ	sc-596		1:500
Cyclin A2	Ms	SANTA CRUZ	sc-271682		1:200
Actin	Rb	SIGMA-ALDRICH	A2066	1:5000	
Secondary antibodies					
Rabbit IgG HRP-conjugated	Go	Jackson	111-035-003	1:7000	
Mouse IgG Alexa 488-conjugated	Go	Molecular Probe	A11029		1:1000
Mouse IgG Alexa 555-conjugated	Go	Molecular Probe	A21424		1:1000
Rabbit IgG Alexa 488-conjugated	Go	Molecular Probe	A11008		1:1000
Rabbit IgG Alexa 555-conjugated	Go	Molecular Probe	A21428		1:1000
Rat IgG Alexa 488-conjugated	Go	Molecular Probe	A11006		1:1000
Rat IgG Alexa 555-conjugated	Go	Molecular Probe	A21434		1:1000

Ms; mouse, Rb; rabbit, Rt; rat, Go; Goat.

Supplementary Table S2

siRNA

Name	Company	Catalog #	Sequence (Sense, 5'->3')
siLIG4-#1	Invitrogen	LIG4HSS180681	CCACACCGUUUAUUUGGACUGUAU
siLIG4-#3	Invitrogen	LIG4HSS106058	AUAUGAAUCAUAAAGCGAGGCUGCC
siR51-#1	Invitrogen	RAD51VHS40453	GCGACUCGCUGAUGAGUUUGGUGUA
siR51-#2	Invitrogen	RAD51VHS40454	CCACCAGACCCAGCUCCUUUAUCAA
siR52-#4	Invitrogen	RAD52_446	CAUUGAGGGUCAUCGGGUAAUUAAU
siR52-#5	ambion	s11746	UGAAGUGGAUUUAACUAAAtt

Primers

Name	Sequence (Sense, 5'->3')
sg-Centromere-f	CACCGAATCTGCAAGTGGATATT
sg-Centromere-r	AAACAATATCCACTTGCAGATTC
sg-Control-f	CACCGACCATGAAGTACCGCATGG
sg-Control-r	AAACCCATGCGGTACTTCATGGTC
hRAD51-N1	TTTGAGCTCATGGCAATGCAGATGCAGCTTGAAGCAAATGC
hRAD51-N2myc	TTTGGATCCGCCGCCACCATGGAACAAAAACTCATCTCAGAAGAGGATCTGGCAATGCAGATGCAGCTTGAAGCAAATGC
hRAD51-C1	TTTGCGGCCGCTCAGTCTTTGGCATCTCCCACTCCATC
hRAD52-N1	TTTCTCGAGATGTCTGGGACTGAGGAAGCAATTCTTGG
hRAD52-N2myc	TTTGAATTCGCCGCCACCATGGAACAAAAACTCATCTCAGAAGAGGATCTGTCTGGGACTGAGGAAGCAATTCTTGG
hRAD52-C1	TTTCTCGAGTTAAGATGGATCATATTTCCTTTTCTTC
